# MFPD: A Multiple Fungal Pathogen Detection Pipeline Across Diverse Habitats

**DOI:** 10.1002/advs.202522660

**Published:** 2026-06-09

**Authors:** Yi Shen, Xinrun Yang, Jiabao Yu, Yaozhong Zhang, Tianjie Yang, Yang Gao, Xiaofang Wang, Alexandre Jousset, Waseem Raza, Fang‐Jie Zhao, Qirong Shen, Gaofei Jiang, Zhong Wei, Yangchun Xu

**Affiliations:** ^1^ Jiangsu Provincial Key Lab for Solid Organic Waste Utilization Key Lab of Organic‐based Fertilizers of China Jiangsu Collaborative Innovation Center of Solid Organic Wastes Educational Ministry Engineering Center of Resource‐Saving Fertilizers Nanjing Agricultural University Nanjing China; ^2^ Genedance GmbH Basel Switzerland; ^3^ National Key Laboratory For Tropical Crop Breeding Sanya Research Institute Institute of Tropical Bioscience and Biotechnology Chinese Academy of Tropical Agricultural Sciences Haikou Hainan China; ^4^ Institute of Vegetable Research Guangxi Academy of Agricultural Sciences Nanning China

**Keywords:** bioinformatics tools, fungal pathogens, ITS sequencing, pathogen detection

## Abstract

Fungal pathogens threaten the health of humans, animals, and plants. ITS sequencing offers an effective approach for detecting fungal pathogens; however, a comprehensive pathogen database and associated tailored pipeline are still lacking. This study introduces the multiple fungal pathogen detection (MFPD) pipeline, which incorporates an accurate and high‐speed sequence alignment algorithm for broad‐habitat pathogen identification. The curated MFPD database includes 95 660 full‐length ITS sequences from 4924 reported fungal pathogen species. In silico experiments show that the full‐length ITS achieves the highest accuracy in pathogen detection (average 99.34%), outperforming both the ITS1 and ITS2 subregions. Benchmarking against existing tools, including FUNGuild, FungalTraits, and ISHAM‐ITS, shows that MFPD achieves the highest F1 scores in mock communities (0.89 for both plant and human–animal pathogens) and detects the broadest spectrum of pathogenic taxa in real samples. In addition to identifying causal pathogens, MFPD can also detect coinfecting pathogens in biological and environmental samples. Together, our work supports pathogen surveillance across diverse sectors, including clinical, agricultural, and livestock systems within a One Health framework.

## Graphical abstract

The MFPD pipeline integrates a comprehensive ITS reference database of fungal pathogens, optimized parameters, and algorithms tailored for both full‐length and subregion sequences that balance accuracy and computational efficiency; it enables high‐throughput, species‐level identification from amplicon sequencing data, supporting large‐scale surveillance across environmental, clinical, and livestock samples.

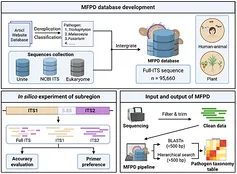

## 1 Introduction

Fungal pathogens represent a major global threat across clinical, agricultural, and environmental settings [1, 2]. Over 2.54 million people worldwide die annually from fungal infections, such as *Cryptococcus neoformans*, *Candida auris*, and *Aspergillus fumigatus* [3]. In agriculture, fungal pathogens, including *Puccinia graminis* (wheat stem rust), *Magnaporthe oryzae* (rice blast), and *Fusarium oxysporum* (banana wilt), cause annual global crop losses of 20%–43% [4]. Coinfections are frequently observed in clinical and agricultural contexts, further worsening disease severity [5, 6, 7]. In addition, increasing evidence suggests that fungal pathogens can transmit across environmental, animal, and human boundaries, highlighting their relevance within the One Health framework [8]. The emergence of antifungal resistance further raises concerns for public health [8]. Moreover, under ongoing climate change, fungi are evolving greater thermotolerance, facilitating the migration of pathogens into previously non‐endemic regions [9]. Such shifts are exacerbated by the ability of dormant spores to persist in the environment and their subsequent transmission across diverse habitats [9]. These dynamics necessitate large‐scale, systematic surveillance to map global pathogen distribution and preempt potential outbreaks [1]. Collectively, these challenges highlight the urgent need for systematic surveillance approaches capable of detecting multiple fungal pathogens across diverse habitats.

Methods for fungal pathogen detection can be broadly classified into culture‐dependent and culture‐independent approaches [10]. Culture‐based methods remain the gold standard for pathogen identification but are time‐consuming, restricted to cultivable pathogens, and generally detect only one pathogen at a time [11], making them impractical for large‐scale sample screening. PCR‐ and probe‐based assays are faster but often constrained by the primer availability and prone to false positives [12]. In contrast to those assays, high‐throughput sequencing methods such as metagenomics and amplicon sequencing offer broad‐spectrum detection, allowing the identification of diverse fungal species simultaneously. However, metagenomics often lacks sufficient sensitivity for identifying fungal species due mainly to their lower abundance relative to bacteria [13] and the lack of a comprehensive fungal reference genome database [14]. Amplicon sequencing provides a cost‐effective, high‐throughput, and high‐resolution strategy that enables simultaneous profiling of multiple fungal taxa across large numbers of samples [15]. Commonly used fungal markers include 18S rRNA, 28S rRNA, and the internal transcribed spacer (ITS) region [15]. Among available genetic markers, ITS regions provide high taxonomic resolution and are therefore the most widely used marker for taxonomic profiling in fungal community analyses [16].

The ITS region comprises two distinct spacers, ITS1 and ITS2, both of which are widely used for fungal identification [17]. Several pipelines and databases, such as FUNGuild [18], FungalTraits [19], and ISHAM‐ITS [20] (referred to as ISHAM hereafter), support fungal pathogen detection through ITS amplicon sequencing. However, these methods either lack associated analytical pipelines [19, 20] or suffer from incomplete coverage of the known pathogen spectrum [19, 20]. Additionally, FUNGuild identifies pathogens solely on the basis of species names without reference sequences [18], resulting in low accuracy. Moreover, the choice of amplified subregion strongly influences taxonomic assignment [21]. Short‐read ITS sequencing is cost‐effective, but the taxonomic resolution is limited and may lead to misclassification between closely related species [22]. Conversely, long‐read ITS sequencing could improve taxonomic resolution and accuracy [23, 24] but is relatively costly and prone to more sequencing errors [23]. Thus, establishing pipelines compatible with both subregion and full‐length ITS sequencing is crucial for advancing fungal pathogen detection.

In this study, we collected reported fungal pathogen species and then integrated existing fungal ITS reference databases to construct a multiple fungal pathogen detection (MFPD) database. This curated database contains 95 660 full‐length ITS sequences representing 4924 reported fungal pathogen species. Then we conducted In silico experiments to determine the optimal ITS subregion and annotation parameters for accurate pathogen detection. Based on optimized parameters, we further developed the user‐friendly pipeline MFPD, which incorporates an accurate and efficient hierarchical sequence search algorithm. Together, MFPD enables the detection of thousands of fungal pathogens across diverse habitats, providing a scalable framework for identifying cryptic coinfections and emerging threats, offering a powerful alternative to single‐target detection methods in large‐scale fungal surveillance.

## 2 Results

### 2.1 Overview of MFPD

**FIGURE 1:**
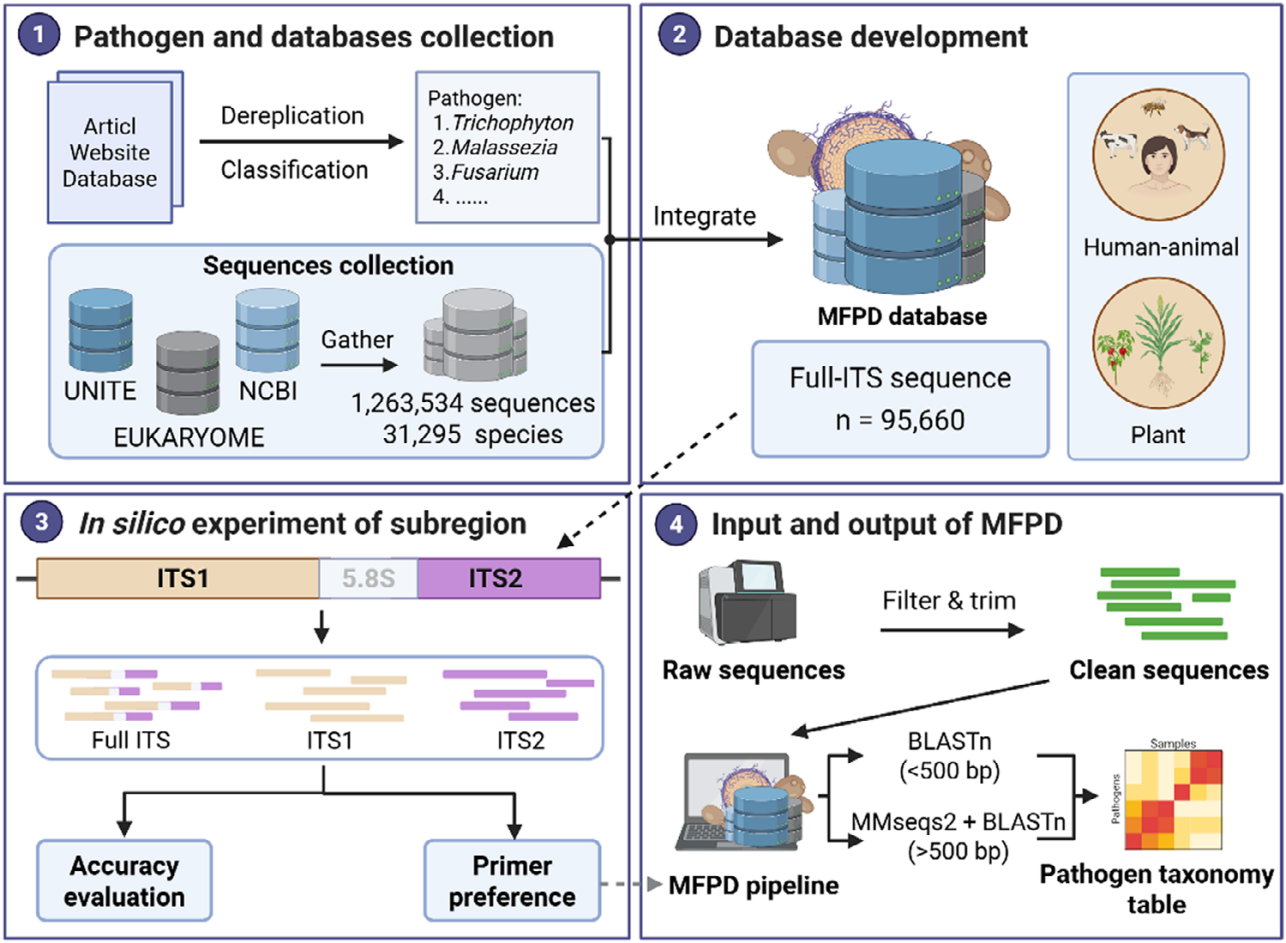
Overview of MFPD. This pipeline offers multiple fungal pathogen detection based on ITS sequencing. (1) Collect fungal pathogen species from published articles, public websites, and databases, and then integrate the existing fungal ITS sequence databases. (2) Fungal pathogen taxa were extracted from existing sequence databases to develop the MFPD database. (3) Evaluate the performance of each ITS subregion under different sequence alignment thresholds. (4) The MFPD pipeline processes ITS sequencing data to generate ASV sequences via the DADA2 package [25]. A feature table of the ASV sequences is then annotated via MMseqs2 [26] and BLASTn [27], resulting in a taxonomy table of fungal pathogens.

We developed MFPD, a broad‐spectrum detection workflow of fungal pathogens based on ITS sequencing. The MFPD development includes four steps: (1) collect fungal pathogen species associated with human‐animal and plant diseases from websites, articles, and databases and integrate commonly used fungal ITS sequence databases, including UNITE, NCBI, and EUKARYOME; (2) extract full‐length ITS sequences of pathogens from existing sequences databases by matching pathogen species to construct the MFPD database, which comprises 95 660 sequences representing 4924 species in total; (3) conduct an In silico test to evaluate the performance of each ITS subregion under different sequence alignment thresholds, leading to recommendation for the optimal ITS subregion and threshold; and (4) the pipeline accepts clean reads obtained after quality filtering, adapter trimming, and paired‐end merging as input and generate a species‐level fungal pathogen annotation ASVs table (Figure 1).

### 2.2 Curated MFPD Pathogen Database

We collected a total of 7831 fungal pathogen species from 66 published articles, public websites, and databases. We subsequently integrated various fungal reference databases, including UNITE, NCBI, and EUKARYOME, yielding 1 263 534 full‐length ITS sequences corresponding to 31 295 fungal species. From this integrated dataset, we extracted pathogen ITS sequences by matching pathogen species names. To eliminate identical sequences from multiple databases, we further removed redundant sequences and kept the most complete variant. Finally resulted in an MFPD database consisting of 95 660 sequences representing 4924 species (Figure S1). Not all reported pathogen species had available full‐length ITS sequences in public databases, so that leaded to a reduced number of species retained in the final database.

**FIGURE 2:**
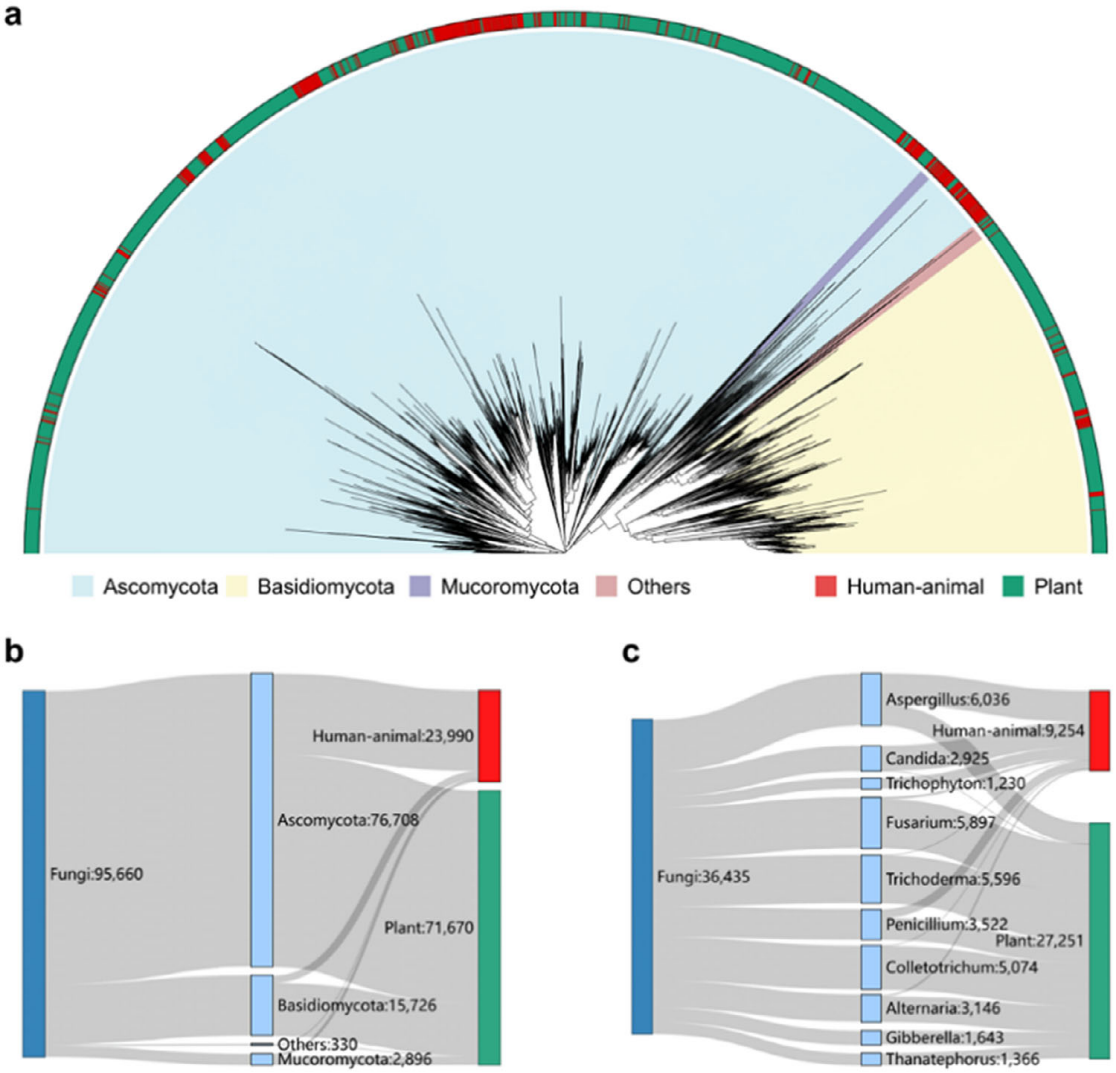
Taxonomic composition of the MFPD database. (a) Phylogenetic tree based on 4,924 reference full‐length ITS sequences from fungal pathogen species. Clade colours correspond to the phylum of the respective species, whereas the outer ring colours represent the host types of the corresponding species. (b) Phylum‐level sequence composition of MFPD, highlighting the three phyla with the largest sequence counts. The remaining categories are grouped under “Others.” (c) The genus‐level sequence composition of MFPD, displaying only the top 10 genera.

In MFPD, these pathogens are grouped into two categories, “human‐animal” and “plant”, according to their infecting host (Figure 2a). Here, “human‐animal” pathogens are pathogens capable of infecting humans, animals, or both [28]. The MFPD database mainly consists of three main phyla: Ascomycota (80.19%, 76 708 sequences), Basidiomycota (20.84%, 15,726 sequences), and Mucoromycota (1.04%, 51 species, 2896 sequences) (Figure 2b and Table S1). Among the total of 4924 pathogen species, 725 (14.72%) were human‐animal pathogens, whereas 4199 (85.28%) were plant pathogens (Figure 2a and Table S1). Specifically, At the genus level, the 10 most represented genera were *Aspergillus* (6.31%, 6,036 sequences), *Fusarium* (6.16%, 5897 sequences), *Trichoderma* (5.85%, 5,596 sequences), *Colletotrichum* (5.30%, 5,074 sequences), *Penicillium* (3.68%, 3522 sequences), *Alternaria* (3.29%, 3,146 sequences), *Candida* (3.06%, 2925 sequences), *Gibberella* (1.72%, 1643 sequences), *Thanatephorus* (1.43%, 1366 sequences), and *Trichophyton* (1.29%, 1230 sequences) (Figure 2c and Table S1). The detailed taxonomic composition of the MFPD database is recorded in Table S1.

### 2.3 Benchmarking of ITS Subregions and Optimization of the Sequence Alignment Algorithm Based on In Silico Experiments

**FIGURE 3:**
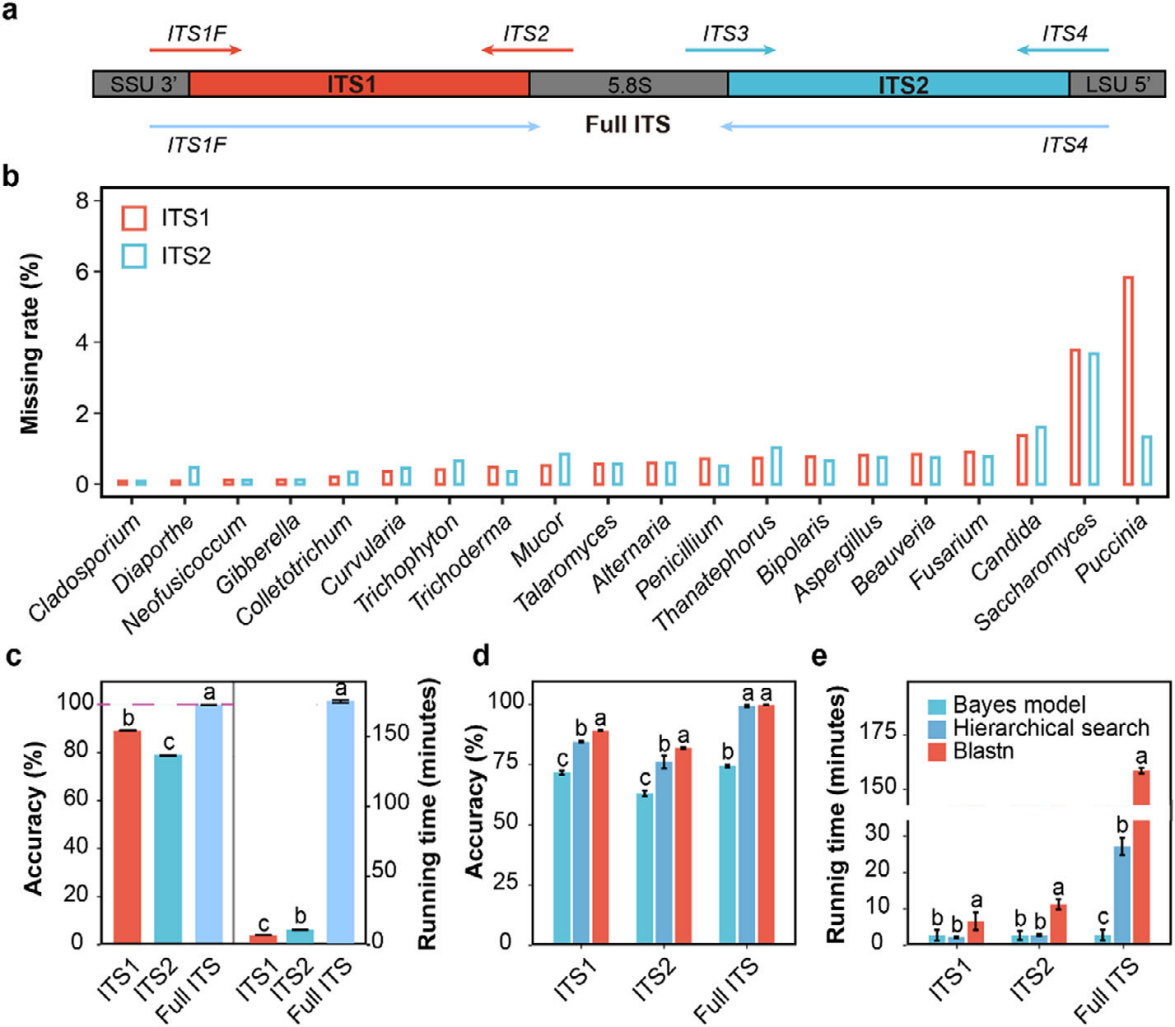
Comparison of the performance between ITS subregions and sequence assignment algorithms via In silico experiments. (a) The ITS region consists of two subregions, ITS1 and ITS2. *ITS1F*, *ITS2*, *ITS3*, and *ITS4* are commonly used primers for ITS1, ITS2, and full‐length ITS amplicon. (b) Missing rates of fungal pathogen sequences across the top 10 genera in the MFPD database under In silico experiments. Different colour bars denote different ITS regions. (c) Taxonomy assignment accuracy (*N = 63*0, n = 210 per subregion, Welch's ANOVA, *F*_(2, 299.56)_ = 461,295, *p < 0.001;* Games–Howell post hoc multiple comparisons, *p < 0.001*) and running time (*N = 630, n = 210* per subregion, Welch's ANOVA, *F*_(2, 352.73)_ = 4,093.2, *p < 0.001;* Games–Howell post hoc multiple comparisons, *p < 0.001*) for ITS subregion sequences across different variable regions of the MFPD database. (d) Taxonomic assignment accuracy of ITS1 (*N* = 90, n = 30 per algorithm, Welch's ANOVA, *F*_(2, 53.20)_ = 22,672, *p < 0.001;* Games–Howell post hoc multiple comparisons, *p < 0.001*), ITS2 (Welch's ANOVA, *F*_(2, 56.25)_ = 12,798, *p < 0.001;* Games–Howell post hoc multiple comparisons, *p < 0.001*), and full‐length ITS (*F*_(2, 39.48)_ = 33,498, *p < 0.001;* Games–Howell post hoc multiple comparisons, *p < 0.001*) regions using different sequence assignment algorithms. (e) Taxonomic assignment running time comparison of ITS1 (*N* = 90, n = 30 per algorithm, Welch's ANOVA, *F*_(2, 41.73)_ = 256.97, *p < 0.001;* Games–Howell post hoc multiple comparisons, *p < 0.001*), ITS2 (Welch's ANOVA, *F*_(2, 46.63)_ = 885.23, *p < 0.001;* Games–Howell post hoc multiple comparisons, *p < 0.001*) and full‐length ITS (Welch's ANOVA, *F*_(2, 39.12)_ = 4,182.6, *p < 0.001;* Games–Howell post hoc multiple comparisons, *p < 0.001*) regions using different sequence assignment algorithms. Different lowercase letters indicate significant differences in detection accuracy across regions. All error bars in this figure denote standard error.

We first extracted the ITS1 and ITS2 sequences from the full‐length ITS sequences with ITSx [29] prediction (Figure 3a) and then compared the missing rates. The missing rate was defined as the proportion of pathogens that cannot be detected by the ITS1 or ITS2 subregion. The full‐length ITS region presented the best pathogen coverage, while the missing rates of ITS1 and ITS2 were both low, at 1.36% across all sequences (Figure S2). However, the ITS1 and ITS2 subregions failed to detect certain strains in some genera, including *Puccinia*, *Saccharomyces*, *Candida*, and *Fusarium*. Among them, *Puccinia* had the highest percentage of missing sequences, with 5.83% of sequences undetected in ITS1 and 1.33% undetected in ITS2 (Figure 3b).

To evaluate the impact of identity thresholds (80%, 85%, 90%, 92%, 95%, 97%, and 99%) on annotation accuracies and running times across ITS1, ITS2, and full‐length ITS regions, we randomly sampled 10 000 sequences from each region, and aligned them to the MFPD database with BLASTn [27], repeated 30 times. The annotation threshold refers to the sequence identity threshold applied for species‐level assignment using BLASTn [27], while annotation accuracy was defined as the proportion of correctly annotated taxa among all tested sequences. Results indicated that while subregion type was the primary driver of accuracy and running time, the identity threshold had a negligible impact on performance (Figure S3a,b). Full‐length ITS achieved the highest accuracy (99.74 ± 0.06%), significantly outperforming ITS1 (89.30 ± 0.02%) and ITS2 (82.06 ± 0.03%) (Figure 3c). However, full‐length ITS was computationally intensive, requiring 172.75 ± 0.75 min for assignment, compared to 5.11 ± 0.15 and 9.63 ± 0.09 min for ITS1 and ITS2, respectively (Figure 3c).

To balance accuracy and efficiency, we then tested the naïve Bayes classifier and the hierarchical search algorithm for the full‐length ITS sequence. The naïve Bayes classifier is a feature‐based classification approach that does not rely on direct sequence alignment, whereas the hierarchical search algorithm adopts a two‐step alignment strategy. Specifically, a guide tree constructed from full‐length reference sequences was first used to perform a coarse placement of the query sequence, followed by a refined alignment within the corresponding clade of the guide tree. In this way, the one‐by‐one alignment performed by BLASTn [27] is replaced by a hierarchical process of approximate localization followed by precise alignment. The naïve Bayes classifier drastically reduced running time across ITS1, ITS2, and full‐length ITS (2.40 ± 1.27, 2.32 ± 1.06, and 2.46 ± 1.27 min, respectively), but its accuracy declined sharply (ITS1: 71.65 ± 0.79%, ITS2: 63.09 ± 1.19%, and full‐length ITS: 74.46 ± 0.60%) (Figure 3d,e). In contrast, the hierarchical search algorithm significantly reduced running time by 86.05% (172.75 ± 0.75 min to 24.09 ± 0.14 min) while maintaining nearly equivalent accuracy for full‐length ITS (99.74 ± 0.06% to 99.34 ± 0.47%). However, this was accompanied by relatively lower accuracy for ITS1 (84.52 ± 0.35%) and ITS2 (76.06 ± 2.64%), despite similarly reduced running times of 2.10 ± 0.03 min and 2.12 ± 0.04 min, respectively (Figure 3d,e). Therefore, we adopted a hybrid strategy: direct BLASTn [27] alignment was used for ITS subregions, while hierarchical search was applied for full‐length sequences. Under this optimized strategy, the ITS1, ITS2, and full‐length ITS regions achieved accuracy of 89.30 ± 0.02%, 82.06 ± 0.03%, and 99.34 ± 0.47% within 5.11 ± 0.15, 9.63 ± 0.09, and 24.09 ± 0.14 min (Figure 3c–e).

To further evaluate which subregion provides higher accuracy for pathogen identification, we calculated the relative accuracy of ITS1 and ITS2 normalized to the full‐length ITS using the aforementioned 30 samples for each subregion, each sample comprising 10,000 randomly selected pathogen sequences. The results demonstrated that ITS1 exhibited higher relative accuracy (Figure S3c) and shorter running time (Figure 3c) compared to ITS2 (Figure 3d,e and Figure S4c). Together, we recommend full‐length ITS sequencing when accuracy is paramount due to its superior accuracy and extensive taxonomic coverage. Conversely, ITS1 sequencing offers a cost‐effective alternative with slightly reduced accuracy for pathogen identification and benefits from its shorter running time.

### 2.4 MFPD Outperforms Existing Tools in Pathogen Detection

**FIGURE 4:**
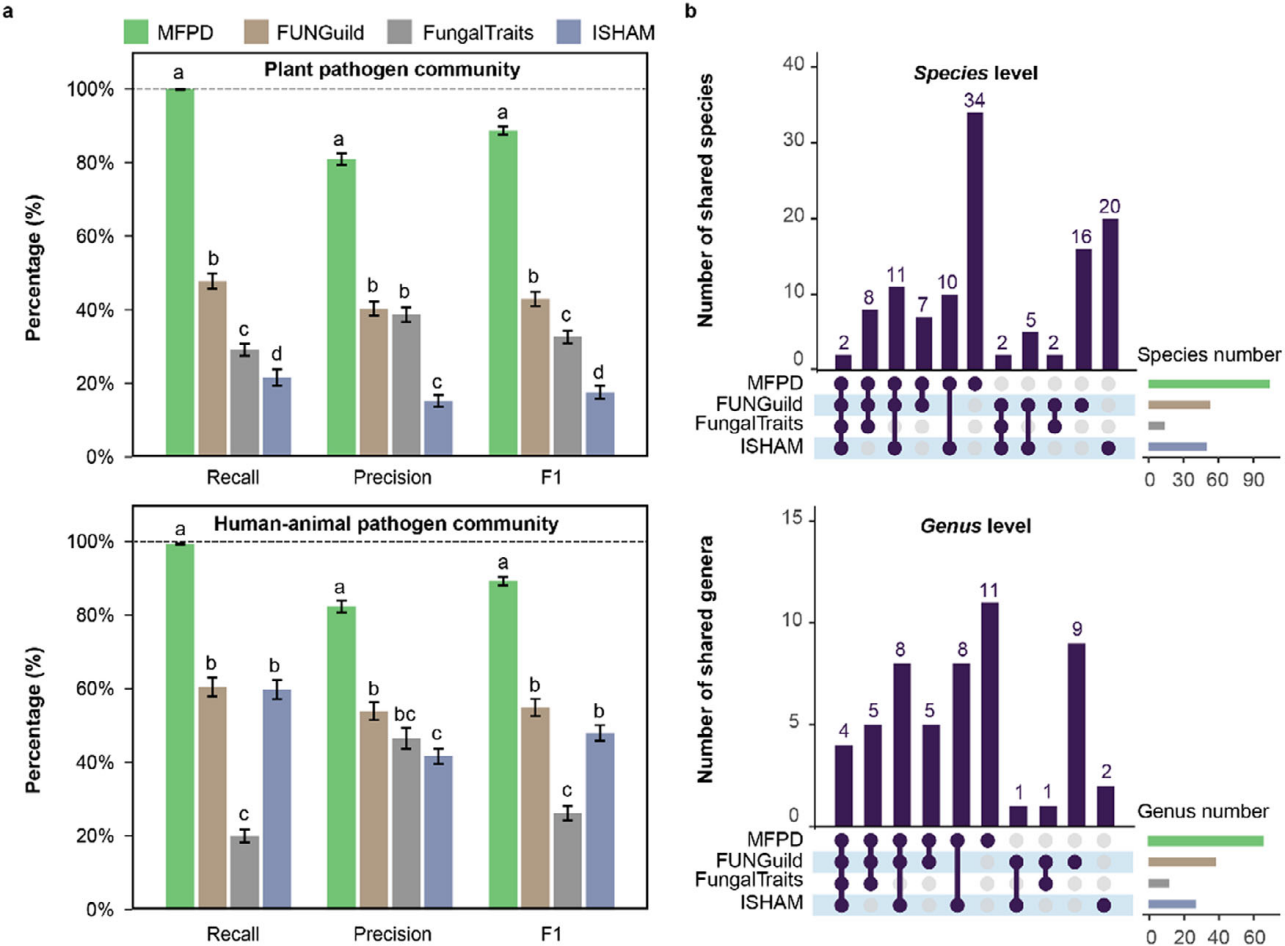
Benchmark of MFPD. (a) Comparison of the recall, precision, and F1 score between MFPD, FUNGuild, FungalTraits, and ISHAM across plant (*N* = 400, *n* = 100 per tool, Welch's ANOVA, *p < 0.001*; Games–Howell post hoc multiple comparisons, *p* < 0.05) and human‐animal (*N* = 400, *n* = 100 per tool, Welch's ANOVA, *p < 0.001*; Games–Howell post hoc multiple comparisons, *p* < 0.05) pathogen mock communities. Different lowercase letters indicate significant differences within each metric. Different colour bars denote different tools. All error bars in this figure denote standard error. (b) Upset diagram illustrating the overlapping and unique fungal pathogens detected by MFPD, FUNGuild, FungalTraits, and ISHAM at the species and genus levels. In the UpSet diagram, connected dots represent subsets of taxa detected by multiple tools, the vertical bars indicate the number of pathogens in each subset, and the horizontal bars show the total number of pathogens detected by each tool.

We first benchmarked MFPD against FUNGuild, FungalTraits, and ISHAM via 100 independent In silico mock communities. Each mock community comprised 20 pathogenic species randomly sampled from MFPD and 10 nonpathogenic species from UNITE (Table S2), with 30 sequences sampled to construct the community. MFPD demonstrated superior performance across all metrics, achieving a recall of 0.99 ± 0.0008, precision of 0.81 ± 0.02, and F1 score of 0.89 ± 0.01 for plant pathogens. While FUNGuild and FungalTraits maintained moderate precision (40–60%), their low recall (below 50%) limited overall performance. Similarly, in the human‐animal pathogen community, MFPD reached the highest recall of 0.99 ± 0.003, precision of 0.82 ± 0.02, and an F1 score of 0.87 ± 0.01 (Figure 4a). This improved performance is likely attributed to the curated fungal pathogen database, the alignment‐based annotation strategy, and the optimized annotation parameters for each variable region.

We then used a published gut microbiome dataset [30] to further evaluate the performance of MFPD in a practical context. As this dataset lacks a defined causal pathogen, the UpSet diagram was used to visualize the shared and unique pathogen taxa identified across different tools. Connected dots denote subsets of taxa detected by multiple tools, with vertical bars indicating subset sizes and horizontal bars showing totals per tool (Figure 4b). MFPD detected the most pathogen species and genera, up to 104 and 65, respectively (Figure 4b). Both MFPD and FUNGuild identified the top 5 fungal pathogen genera (*Candida*, *Fusarium*, *Rhizopus*, *Cutaneotrichosporon*, and *Vanrija*), whereas FungalTraits and ISHAM, which primarily focus on plant or human‐animal pathogens, failed to detect all these genera (Figure S4a). Notably, *Candida*, *Fusarium*, and *Vanrija* have been consistently reported as dominant and disease‐associated genera in previous studies [30]. At the species level, MFPD and FUNGuild consistently detected dominant pathogenic species, including *Candida albicans*, *Fusarium commune*, and *Rhizopus arrhizus* (Figure S4b,c).

### 2.5 Validation of MFPD Using Reported Samples with Known Causal Pathogens

**FIGURE 5:**
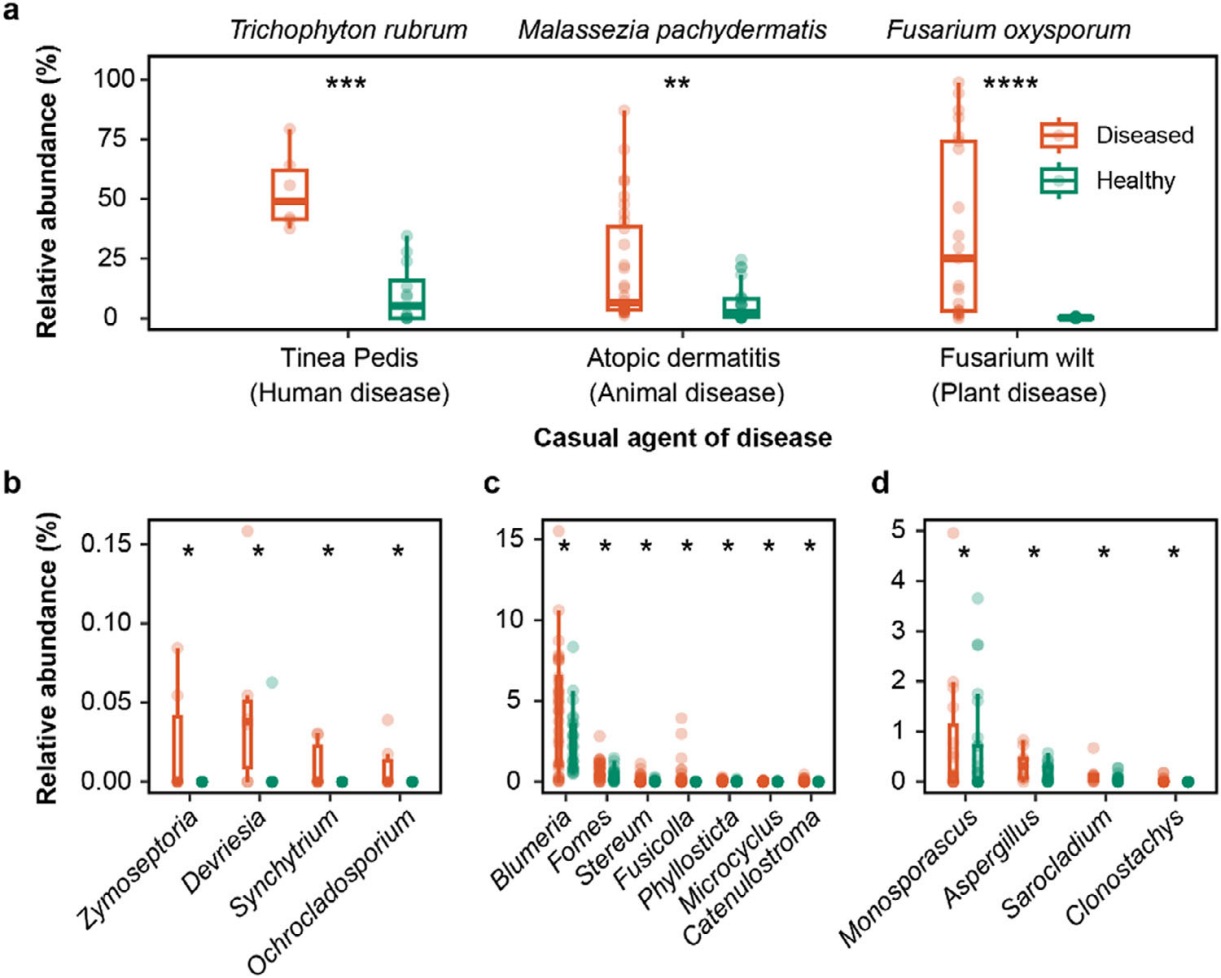
Detection of fungal pathogens in healthy and diseased samples by MFPD. (a) Variations in the relative abundance of causal pathogens between healthy and diseased samples across human (*N* = 18), animal (*N* = 56), and plant rhizosphere (*N* = 24). (b–d) Relative abundances of other potential genera enriched in the diseased group in the human (b), animal (c), and plant rhizosphere (d) samples. Statistical analyses were performed via the Wilcoxon rank‐sum test (^*^ *p* < 0.05; ^**^ *p* < 0.01; ^***^ *p* < 0.001; ^****^ *p* < 0.0001).

To further evaluate the performance of MFPD on published datasets with known causal fungal pathogens, we applied MFPD to paired diseased and healthy datasets from humans, animals, and plants. These datasets included human tinea pedis caused by *Trichophyton rubrum* [31], dog atopic dermatitis caused by *Malassezia pachydermatis* [32], and watermelon Fusarium wilt caused by *Fusarium oxysporum* [33]. In all the cases, the relative abundances of the causal pathogens were significantly higher in diseased samples than in healthy ones (Wilcoxon rank‐sum test, *p* < 0.01; Figure 5a and Figure S5a–c). Pathogen diversity was found to be reduced in the diseased samples (Wilcoxon rank‐sum test, *p* < 0.05; Figure S6d–f). Additionally, MFPD was able to detect potential coinfecting pathogens (Figure 5b–d). For example, *Monosporascus* was highly abundant in diseased plant samples (Wilcoxon rank‐sum test, *p* < 0.05), which commonly causes root rot and vine decline in melon crops [34]. Importantly, while these signals suggest a potential synergistic relationship, they represent co‐occurrence patterns rather than definitive evidence of cooperative infection.

## 3 Discussion

In this study, we developed an MFPD pipeline based on ITS sequencing to address the need for broad‐spectrum fungal surveillance across biological and environmental samples, providing a scalable screening tool for identifying emerging threats and cryptic co‐infections in complex habitats. The key features of our research are as follows: (1) a relatively comprehensive database containing 96 660 reference sequences from 4924 fungal pathogen species labelled with host types; (2) a well‐tuned pipeline applicable to both short‐ and full‐length ITS sequences, with recommended ITS subregions and taxonomic annotation thresholds; (3) accurate pathogen identification across diverse habitats; and (4) the detection of potential coinfecting pathogens.

Current fungal taxonomic reference databases are incomplete and often fragmented, differing from the well‐organized bacterial database [35]. To address this, we integrated reported fungal pathogen species and compiled full‐length ITS sequences from commonly used databases to establish a more comprehensive fungal pathogen reference database. We initially collected 7,831 fungal pathogenic species, but nearly 3000 species could not be retrieved from existing databases, underscoring the gap between taxonomic records and available reference sequences. Future efforts should prioritize generating reference sequences for these underrepresented species to bridge this gap. Moreover, plant pathogens were more dominant in the fungal pathogen database, which differs from our previously constructed multiple bacterial pathogen database, MBPD [36]. This pattern may be due to the stronger emphasis on human‐animal pathogens in bacteria and is also consistent with more severe crop loss caused by fungal pathogens in agricultural practices [37].

The accuracy of pathogen identification was affected by the choice of ITS subregion but not the sequence alignment thresholds. This may be due to the strong specificity of the ITS region [38]. Among subregions, ITS1 provides comparable coverage to ITS2 while achieving higher accuracy and shorter running time, representing an efficient alternative to full‐length ITS. To balance accuracy and efficiency in full‐length ITS sequence alignment, we adopted a hierarchical search strategy. Specifically, we used CD‐HIT [39] to conduct a guide tree from the MFPD database based on sequence identity. ASV sequences were rapidly mapped to the clades of the tree with MMseqs2 [26], followed by precise alignment to the leaf nodes within each clade using BLASTn [27] to identify the closest reference sequence and taxonomic assignment. This strategy reduces computational cost [40] and markedly reduces running time while maintaining high accuracy. QIIME 2 [41] naïve Bayesian model classifier did not achieve high accuracy in our study, likely due to large variations in sequence length [41] and the highly uneven distribution of reference sequences across taxa [42].

Next, we benchmark MFPD against FUNGuild [18], FungalTraits [19], and ISHAM [20] via both mock communities and real biological samples. In the mock community test, MFPD achieved the highest F1 scores. The superior performance of MFPD was attributable to its integrated design, combining a curated database of 96 660 full‐length ITS sequences with a tailored analysis pipeline. This holistic framework ensures broad‐spectrum detection and high taxonomic resolution, whereas the specific algorithm optimizes computational efficiency. In real clinical data, both MFPD and FUNGuild [18] identified the abundant pathogen genera, whereas FungalTraits [19] and ISHAM [20] failed to capture them. This difference is mainly due to pathogen coverage: MFPD and FUNGuild cover both plant and human–animal pathogens [18], whereas FungalTraits and ISHAM are restricted to only plant or human–animal pathogens [19, 20]. Importantly, MFPD successfully captures co‐occurrence signals between causal and coinfecting pathogens associated with disease across human gut, animal skin, and rhizosphere habitats. For instance, in the watermelon wilt case, MFPD identified *Monosporascus* alongside *Fusarium oxysporum*, revealing synergistic pathogenic relationships that traditional qPCR or culture‐based methods might overlook. These findings demonstrate its potential in revealing major and cryptic pathogenic factors in complex settings, such as fungal systemic infection, animal infections, and crop diseases caused by unknown pathogens.

There are still limitations and challenges in pathogen detection via ITS sequencing. First, ITS sequencing lacks the functional resolution to distinguish pathogenic strains from non‐pathogenic or pathogenic ones within the same species. For example, a fungal pathogen, *Fusarium oxysporum*, exhibits high levels of host specialization [43], which cannot be differentiated solely by ITS sequences. Second, high relative abundance or co‐occurrence signals indicate a pathogenic potential rather than direct evidence of active disease or coinfection. Despite these limitations, MFPD can serve as a useful tool to prioritize candidate pathogens, thereby narrowing the scope for downstream pathogen screening and experimental validation (e.g., fulfillment of Koch's postulates or strain‐level genomics). Third, shared ITS sequences among species complicate identification within certain genera when short‐read ITS sequencing is used [17, 44]. For example, economically important pathogenic genera such as *Cladosporium*, *Penicillium*, and *Fusarium* contain species with similar fragments in the ITS region, which might lead to misclassification [17]. Full‐length sequencing can offer increased taxonomic resolution to address this limitation [17, 20]. Although still more expensive than short‐read sequencing, its cost is steadily decreasing, and it is expected to become increasingly feasible [23].

## 4 Conclusion

We developed the MFPD pipeline based on ITS sequencing, enabling the detection of both causal and coinfecting pathogens across multiple habitats. MFPD is supported by a comprehensive database containing 95 660 reference sequences from 4 924 fungal pathogen species. The benchmarking results demonstrate that MFPD outperforms existing tools, achieving particularly high accuracy in long‐read sequence identification. We anticipate that MFPD can be widely applied for fungal pathogen monitoring, assessment, and diagnosis in clinical, agricultural, and veterinary settings, aligning with the One Health perspective.

## 5 Methods

### 5.1 Pathogen Database Construction

We compiled data on fungal pathogen species and their host ranges from 66 studies and databases (Tables S3 and S4), including the Pathogen Host Interactions (PHI‐base) [45], the National Pathogen Resources Center (https://www.nprc.org.cn/), and the Global Catalogue of Pathogens. Host ranges are classified into two categories: “Plant” and “Human‐Animal”. The latter category includes pathogens capable of infecting humans, animals, or zoonotic [28]. Next, we integrated annotated fungal sequences from multiple databases: UNITE [46], EUKARYOME [47], and the NCBI ITS Project (https://ftp.ncbi.nlm.nih.gov/genomes/TARGET/ITS_rRNA/), resulting in a total of 1,263,534 fungal sequences. Then, we extracted the sequences of fungal pathogen species and used CD‐HIT v4.8.1 [39] with the parameter “‐c 1.0” to remove redundant sequences and keep the longest one. Finally, the MFPD pathogen database contained 95,660 sequences representing 4,924 fungal pathogen species (Table S5). Phylogenetic analysis was performed by aligning sequences with MAFFT v7.52 [48] and constructing a maximum likelihood tree via FastTree v2.1 [49]. The resulting tree was visualized via the iTOL web‐based tool [50].

### 5.2 Parameter and Sequence Alignment Algorithm Optimization on ITS Subregions and Annotation Thresholds

We employed ITSx v1.1.3 [29] to extract ITS subregions from the MFPD database. To evaluate taxonomic assessment variations across subregion sequences under different sequence alignment thresholds, we randomly sampled 10 000 sequences from each ITS subregion dataset via seqkit v2.6.1 [51]. These sequences were then aligned with the MFPD database via BLASTn v2.17.0+ [27] at varying sequence identity thresholds (80%, 85%, 90%, 92%, 95%, 97% and 99%). Considering the cost‐effectiveness and widespread use of subregions, the relative accuracy rate was used to assess the optimal performance of each subregion for pathogen identification:

$$Accuracy\;rate=\frac{\left(accuracy_{full-length}-accuracy_{subregions}\right)}{accuracy_{full-length}}$$

Based on the long running time of directly BLASTn [27] alignment, we compared different algorithms to identify a more cost‐effective option, including BLASTn [27], the naïve Bayes classifier, and a hierarchical search strategy. BLASTn [27] alignments were performed with the parameter “‐perc_identity 97”. The naïve Bayes classifier was trained and applied using QIIME 2 [41] version amplicon‐2025.7 with default settings. The hierarchical search specifically includes these steps. First, the MFPD database was clustered using CD‐HIT v4.8.1 [39] with the parameter “‐c 0.95” to generate representative reference sequences. Query sequences were aligned to these representative reference sequences using MMseqs2 v18.8 cc5c [26] with the parameter “–min‐seq‐id 0.9” to identify candidate clusters. Finally, within each candidate cluster, BLASTn v2.17.0+ [27] was employed with the parameter “‐perc_identity 97 ‐evalue 1e‐5” to determine the best‐matching reference sequence.

### 5.3 In Silico Mock Community Construction

We randomly selected 10 nonpathogenic species and 20 plant pathogen species from the UNITE [46] and the MFPD database, respectively (Table S2). For each selected species, we randomly extracted 10 corresponding sequences from the MFPD database, creating a sequence pool that included 300 sequences from 30 fungal species. We then sampled 30 sequences from the pool to construct a mock plant pathogen community and analysed it with MFPD, FUNGuild, FungalTraits, and ISHAM to calculate the recall, precision, and F1 score; this process was repeated 100 times. We also processed the same data as above to construct a human‐animal pathogen mock community to evaluate database performance.

### 5.4 Performance of MFPD on Real Biological and Environmental Samples

To compare the performance of the MFPD and FUNGuild pipelines [18], we downloaded reported human gut datasets of systemic lupus erythematosus patients [30] from a public database for species identification testing (NCBI Accession Code: PRJNA900285). This dataset had previously been tested via the FUNGuild [18]. For subsequent analysis, we filtered out ASVs with an average number of reads less than 10 to avoid random errors caused by sequencing. Moreover, to evaluate the performance of MFPD on real pathogenic datasets with known causal fungal pathogens, we downloaded three published paired diseased and control samples, one each from human [31] (NCBI Accession Code: SRP167680), animal [32] (NCBI Accession Code: PRJEB20808), and environment [33] (NCBI Accession Code: PRJNA643411) sources. The raw sequences were processed via fastp v0.23.4 [52] to filter out low‐quality sequences and trim barcodes. Paired‐end sequences were then merged via VSEARCH v2.29.1 [53], trimmed, and denoised, and chimeric sequences were removed via the DADA2 [25] package, resulting in clean reads. For taxonomic annotation, since FUNGuild [18] and FungalTraits [19] do not provide reference sequences, the clean reads were classified against the widely recognized UNITE [46] database. MFPD and ISHAM [20] contain reference sequences and directly perform taxonomic annotation. Finally, pathogen taxonomy files were generated via the scripts provided in the MFPD pipeline.

### 5.5 Data Processing

The raw Illumina sequencing data were processed with fastp v0.23.4 [52] to filter low‐quality sequences, remove barcodes and primers, and then merged with VSEARCH v2.29.1 [53]. The merged sequences were processed via the DADA2 [25] package for trimming, denoising, and chimeric sequence removal. For taxonomic assignment, considering the high computational cost of BLASTn v2.2.30 [27] for full‐length sequences, we adopted a hybrid strategy: direct BLASTn v2.17.0+ [27] with parameters “‐perc_identity 0.97 ‐evalue 1e‐5” against the MFPD database was applied for subregions, while a hierarchical search strategy was used for full‐length sequences.

### 5.6 Statistical Analysis

Pathogen abundance values were log‐transformed to improve visualization of differences. All other results were presented using the original values. Data were presented as mean ± standard deviation (SD). Due to unequal variances and a large sample size, Welch's ANOVA followed by Games–Howell post hoc multiple comparisons (two‐sided) were used to determine the statistical significance of multiple comparisons. For comparisons of classification accuracy between the ITS1 and ITS2 subregions, the Wilcoxon rank‐sum test (two‐sided) was applied because the data were highly concentrated and did not satisfy the assumption of normality. For comparisons of the relative abundances of causal and potential pathogens, the Wilcoxon rank‐sum test (two‐sided) was also used due to the small sample size and possible deviations from normality. The Wilcoxon rank‐sum test (two‐sided) was used to compare pathogen abundance and the Shannon diversity index between diseased and healthy samples because normality could not be assumed. Statistical significance was defined as *p* < 0.05. All analyses were conducted using R version 4.3.3. Shannon diversity was calculated using the *vegan* package. Welch's ANOVA was performed using the *stats* package, and Games–Howell post hoc tests and Wilcoxon rank‐sum tests were conducted using the *rstatix* package. The phylogenetic tree was generated using Interactive Tree Of Life (iTOL) [50]. Plots were generated using the *ggplot2* package.

## Author Contributions

Conceptualization: Y.S, X.Y, G.J, and Y.X; Resources: G.J, Q.S, Z.W, and Y.X; Methodology: Y.S, X.Y, J.Y, Y.Z, A.J, and F.Z; Data curation: Y.S, X.Y, Y.Z, X.W, and G.J; Formal analysis: Y.S, X.Y, J.Y, T.Y, Y.G, and A.J; Funding acquisition: Q.S, G.J, Z.W, and Y.X; Investigation: Y.S, X.Y, J.Y, X.W, A.J, F.Z, Z.W, and G.J; Project Administration: Y.S, X.Y, J.Y, G.J, Z.W, and Y.X; Supervision: G.J, Z.W, and Y.X; Software: Y.S, X.Y, and Y.Z; Visualization: Y.S, X.Y, Y.G, G.J; Writing – original draft: Y.S, X.Y, J.Y, Y.Z, T.Y, Y.G, and X.W; Writing – review & editing: Y.S, X.Y, J.Y, T.Y, Y.G, X.W, A.J, F.Z, Q.S, G.J, and Y.X.

## Conflicts of Interest

The authors declare no conflict of interest.

## Supporting information




**Supporting File 1**: advs75979‐sup‐0001‐Figure S1‐S5.docx.

**Supporting File 2**: advs75979‐sup‐0002‐Table S1‐S5.xlsx.

## Data Availability

MFPD is publicly available on GitHub (https://github.com/LorMeBioAI/MFPD). The Illumina sequence reads used in this study are publicly available at the NCBI Sequence Read Archive under accession number SRP167680 and the European Nucleotide Archive (ENA) database [54] under accession numbers PRJNA900285, PRJEB20808, and PRJNA643411. Supplementary data related to this article can be found on the journal's website.

## References

[1] A. Yiallouris , Z. D. Pana , and G. Marangos , “Fungal Diversity in the Soil Mycobiome: Implications for One Health,” One Health 18 (2024): 100720, 10.1016/j.onehlt.2024.100720. 38699438 PMC11064618

[2] M. C. Fisher , S. J. Gurr , and C. A. Cuomo , “Threats Posed by the Fungal Kingdom to Humans, Wildlife, and Agriculture,” MBio 11, no. 3 (2020): e00449, 10.1128/mBio.00449-20. 32371596 PMC7403777

[3] D. W. Denning , “Global Incidence and Mortality of Severe Fungal Disease,” The Lancet Infectious Diseases 24, no. 7 (2024): e428–e438, 10.1016/S1473-3099(23)00692-8. 38224705

[4] E. Stukenbrock and S. Gurr , “Address the Growing Urgency of Fungal Disease in Crops,” Nature 617, no. 7959 (2023): 31–34, 10.1038/d41586-023-01465-4. 37130937

[5] E. C. Griffiths , A. B. Pedersen , A. Fenton , and O. L. Petchey , “The Nature and Consequences of Coinfection in Humans,” Journal of Infection 63, no. 3 (2011): 200–206, 10.1016/j.jinf.2011.06.005. 21704071 PMC3430964

[6] F. Salazar , E. Bignell , G. D. Brown , P. C. Cook , and A. Warris , “Pathogenesis of Respiratory Viral and Fungal Coinfections,” Clinical Microbiology Reviews 35, no. 1 (2022): e00094, 10.1128/CMR.00094-21. 34788127 PMC8597983

[7] L. Lansbury , B. Lim , V. Baskaran , and W. S. Lim , “Co‐infections in People with COVID‐19: a Systematic Review and Meta‐analysis,” Journal of Infection 81, no. 2 (2020): 266–275, 10.1016/j.jinf.2020.05.046. 32473235 PMC7255350

[8] I. D. Iliev , G. D. Brown , and P. Bacher , “Focus on Fungi,” Cell 187, no. 19 (2024): 5121–5127, 10.1016/j.cell.2024.08.016. 39303681 PMC11722117

[9] D. Seidel , S. Wurster , and J. D. Jenks , “Impact of Climate Change and Natural Disasters on Fungal Infections,” The Lancet Microbe 5, no. 6 (2024): e594–e605, 10.1016/S2666-5247(24)00039-9. 38518791

[10] N. Luchi , R. Ioos , and A. Santini , “Fast and Reliable Molecular Methods to Detect Fungal Pathogens in Woody Plants,” Applied Microbiology and Biotechnology 104, no. 6 (2020): 2453–2468, 10.1007/s00253-020-10395-4. 32006049 PMC7044139

[11] J. A. Tomlinson , M. Dickinson , E. Hobden , S. Robinson , P. M. Giltrap , and N. Boonham , “A Five‐minute DNA Extraction Method for Expedited Detection of Phytophthora ramorum Following Prescreening Using Phytophthora spp. Lateral Flow Devices,” Journal of Microbiological Methods 81, no. 2 (2010): 116–120, 10.1016/j.mimet.2010.02.006. 20171248

[12] S. Aslam , A. Tahir , M. F. Aslam , M. W. Alam , A. A. Shedayi , and S. Sadia , “Recent Advances in Molecular Techniques for the Identification of Phytopathogenic Fungi—A Mini Review,” Journal of Plant Interactions 12, no. 1 (2017): 493–504, 10.1080/17429145.2017.1397205.

[13] G. E. De Albuquerque , B. S. Moda , M. S. Serpa , et al., “Evaluation of Bacteria and Fungi Dna Abundance in human Tissues,” Genes 13, no. 2 (2022): 237, 10.3390/genes13020237. 35205282 PMC8872151

[14] E. Avershina , A. I. Qureshi , H. C. Winther‐Larsen , et al., “Challenges in Capturing the Mycobiome from Shotgun Metagenome Data: Lack of Software and Databases,” Microbiome 13, no. 1 (2025): 66, 10.1186/s40168-025-02048-3. 40055808 PMC11887097

[15] L. Ma , F. A. Jakobiec , and T. P. Dryja , “A Review of Next‐Generation Sequencing (NGS): Applications to the Diagnosis of Ocular Infectious Diseases,” Seminars in Ophthalmology 34, no. 4 (2019): 223–231, 10.1080/08820538.2019.1620800. 31170015

[16] A. V. Ivanov , I. V. Safenkova , A. V. Zherdev , and B. B. Dzantiev , “The Potential Use of Isothermal Amplification Assays for in‐field Diagnostics of Plant Pathogens,” Plants 10, no. 11 (2021): 2424, 10.3390/plants10112424. 34834787 PMC8621059

[17] C. L. Schoch , K. A. Seifert , and S. Huhndorf , “Nuclear Ribosomal Internal Transcribed Spacer (its) Region as a Universal Dna Barcode Marker for Fungi,” Proceedings of the National Academy of Sciences 109, no. 16 (2012): 6241–6246, 10.1073/pnas.1117018109. PMC3341068 22454494

[18] N. H. Nguyen , Z. Song , and S. T. Bates , “FUNGuild: an Open Annotation Tool for Parsing Fungal Community Datasets by Ecological guild,” Fungal Ecology 20 (2016): 241–248, 10.1016/j.funeco.2015.06.006.

[19] S. Põlme , K. Abarenkov , and R. Henrik Nilsson , “FungalTraits: a User‐friendly Traits Database of Fungi and Fungus‐Like Stramenopiles,” Fungal Diversity 105, no. 1 (2020): 1–16, 10.1007/s13225-020-00466-2.

[20] L. Irinyi , C. Serena , and D. Garcia‐Hermoso , “International Society of Human and Animal Mycology (ISHAM)‐ITS Reference DNA Barcoding Database—The Quality Controlled Standard Tool for Routine Identification of human and Animal Pathogenic Fungi,” Medical Mycology 53, no. 4 (2015): 313–337, 10.1093/mmy/myv008. 25802363

[21] L. Tedersoo , M. Bahram , and L. Zinger , “Best Practices in Metabarcoding of Fungi: from Experimental Design to Results,” Molecular Ecology 31, no. 10 (2022): 2769–2795, 10.1111/mec.16460. 35395127

[22] P. Chen , W. Sun , and Y. He , “Comparison of the next‐generation Sequencing (NGS) Technology with Culture Methods in the Diagnosis of Bacterial and Fungal Infections,” Journal of Thoracic Disease 12, no. 9 (2020): 4924–4929, 10.21037/jtd-20-930. 33145066 PMC7578456

[23] C. Scarano , I. Veneruso , R. R. De Simone , G. Di Bonito , A. Secondino , and V. D'Argenio , “The Third‐generation Sequencing Challenge: Novel Insights for the Omic Sciences,” Biomolecules 14, no. 5 (2024): 568, 10.3390/biom14050568. 38785975 PMC11117673

[24] L. Zhan , C. Gui , W. Wei , J. Liu , and B. Gui , “Third Generation Sequencing Transforms the Way of the Screening and Diagnosis of Thalassemia: a Mini‐review,” Frontiers in Pediatrics 11 (2023): 1199609, 10.3389/fped.2023.1199609. 37484768 PMC10357962

[25] B. J. Callahan , P. J. McMurdie , M. J. Rosen , A. W. Han , A. J. A. Johnson , and S. P. Holmes , “DADA2: High‐resolution Sample Inference from Illumina Amplicon Data,” Nature Methods 13, no. 7 (2016): 581–583, 10.1038/nmeth.3869. 27214047 PMC4927377

[26] M. Steinegger and J. Söding , “MMseqs2 enables Sensitive Protein Sequence Searching for the Analysis of Massive Data Sets,” Nature Biotechnology 35, no. 11 (2017): 1026–1028, 10.1038/nbt.3988. 29035372

[27] C. Camacho , G. Coulouris , and V. Avagyan , “BLAST+: Architecture and Applications,” BMC Bioinformatics [Electronic Resource] 10 (2009): 421, 10.1186/1471-2105-10-421. 20003500 PMC2803857

[28] X. Yang , C. Li , and D. Ouyang , “High Microbiome Diversity Constricts the Prevalence of human and Animal Pathogens in the Plant Rhizosphere Worldwide,” One Earth 7, no. 7 (2024): 1301–1312, 10.1016/j.oneear.2024.06.005.

[29] J. Bengtsson‐Palme , M. Ryberg , and M. Hartmann , “Improved Software Detection and Extraction of ITS1 and ITS 2 from Ribosomal ITS Sequences of Fungi and Other Eukaryotes for Analysis of Environmental Sequencing Data,” Methods in Ecology and Evolution 4, no. 10 (2013): 914–919, 10.1111/2041-210X.12073.

[30] P. Yang , R. Xu , and F. Chen , “Fungal Gut Microbiota Dysbiosis in Systemic Lupus Erythematosus,” Frontiers in Microbiology 14 (2023): 1149311, 10.3389/fmicb.2023.1149311. 37089568 PMC10115219

[31] X. Liu , J. Tan , and H. Yang , “Characterization of Skin Microbiome in Tinea Pedis,” Indian Journal of Microbiology 59, no. 4 (2019): 422–427, 10.1007/s12088-019-00816-y. 31762504 PMC6842383

[32] S. Chermprapai , T. H. A. Ederveen , and F. Broere , “The Bacterial and Fungal Microbiome of the Skin of Healthy Dogs and Dogs with Atopic Dermatitis and the Impact of Topical Antimicrobial Therapy, an Exploratory Study,” Veterinary Microbiology 229 (2019): 90–99, 10.1016/j.vetmic.2018.12.022. 30642603

[33] L. Xu , M. Nicolaisen , J. Larsen , R. Zeng , S. Gao , and F. Dai , “Pathogen Infection and Host‐Resistance Interactively Affect Root‐Associated Fungal Communities in Watermelon,” Frontiers in Microbiology 11 (2020): 605622, 10.3389/fmicb.2020.605622. 33424807 PMC7793699

[34] S. A. Marquez , J. Jifon , K. M. Crosby , et al., “Heterosis of Vine Decline Disease Resistance Caused by the Fungus Monosporascus Cannonballus in Melons (cucumis melo L),” Agricultural Sciences 14, no. 05 (2023): 629–635, 10.4236/as.2023.145042. PMC9985897 36883060

[35] C. Quast , E. Pruesse , and P. Yilmaz , “The Silva Ribosomal Rna Gene Database Project: Improved Data Processing and Web‐based Tools,” Nucleic Acids Research 41 (2012): D590–D596, 10.1093/nar/gks1219. 23193283 PMC3531112

[36] X. Yang , G. Jiang , and Y. Zhang , “MBPD: a Multiple Bacterial Pathogen Detection Pipeline for One Health Practices,” IMeta 2, no. 1 (2023): 82, 10.1002/imt2.82. PMC10989770 38868336

[37] P. A. Nazarov , D. N. Baleev , M. I. Ivanova , L. M. Sokolova , and M. V. Karakozova , “Infectious Plant Diseases: Etiology, Current Status, Problems and Prospects in Plant Protection,” Acta Naturae 12, no. 3 (2020): 46–59, 10.32607/actanaturae.11026. 33173596 PMC7604890

[38] H. Mbareche , M. Veillette , G. Bilodeau , and C. Duchaine , “Comparison of the Performance of its1 and its2 as Barcodes in Amplicon‐based Sequencing of Bioaerosols,” PeerJ 8 (2020): 8523, 10.7717/peerj.8523. PMC7032056 32110484

[39] L. Fu , B. Niu , Z. Zhu , S. Wu , and W. Li , “CD‐HIT: Accelerated for Clustering the next‐generation Sequencing Data,” Bioinformatics 28, no. 23 (2012): 3150–3152, 10.1093/bioinformatics/bts565. 23060610 PMC3516142

[40] F. Corpet , “Multiple Sequence Alignment with Hierarchical Clustering,” Nucleic Acids Research 16, no. 22 (1988): 10881–10890, 10.1093/nar/16.22.10881. 2849754 PMC338945

[41] N. A. Bokulich , B. D. Kaehler , and J. R. Rideout , “Optimizing Taxonomic Classification of Marker‐gene Amplicon Sequences with qiime 2's q2‐feature‐classifier Plugin,” Microbiome 6, no. 1 (2018): 90, 10.1186/s40168-018-0470-z. 29773078 PMC5956843

[42] B. D. Kaehler , N. A. Bokulich , D. McDonald , R. Knight , J. G. Caporaso , and G. A. Huttley , “Species Abundance Information Improves Sequence Taxonomy Classification Accuracy,” Nature Communications 10, no. 1 (2019): 4643, 10.1038/s41467-019-12669-6. PMC6789115 31604942

[43] T. Satyanarayana , S. K. Deshmukh , and B. N. Johri , eds., Developments in fungal biology and applied mycology (Springer, 2017), 10.1007/978-981-10-4768-8.

[44] R. H. Nilsson , E. Kristiansson , M. Ryberg , N. Hallenberg , and K.‐H. Larsson , “Intraspecific Its Variability in the kingdom Fungi as Expressed in the International Sequence Databases and Its Implications for Molecular Species Identification,” Evolutionary Bioinformatics 4 (2008): EBOS653, 10.4137/EBO.S653. PMC2614188 19204817

[45] M. Urban , A. Cuzick , J. Seager , et al., “PHI‐base in 2022: a Multi‐species Phenotype Database for Pathogen–host Interactions,” Nucleic Acids Research 50, no. D1 (2022): D837–D847, 10.1093/nar/gkab1037. 34788826 PMC8728202

[46] K. Abarenkov , R. H. Nilsson , and K.‐H. Larsson , “The UNITE Database for Molecular Identification and Taxonomic Communication of Fungi and Other Eukaryotes: Sequences, Taxa and Classifications Reconsidered,” Nucleic Acids Research 52 (2024): D791–D797, 10.1093/nar/gkad1039. 37953409 PMC10767974

[47] L. Tedersoo , M. S. Hosseyni Moghaddam , and V. Mikryukov , “EUKARYOME: the rrna Gene Reference Database for Identification of all Eukaryotes,” Database 2024 (2024): baae043, 10.1093/database/baae043. 38865431 PMC11168333

[48] J. Rozewicki , S. Li , and K. M. Amada , “MAFFT‐dash: Integrated Protein Sequence and Structural Alignment,” Nucleic Acids Research 47 (2019): gkz342, 10.1093/nar/gkz342. PMC6602451 31062021

[49] M. N. Price , P. S. Dehal , and A. P. Arkin , “FastTree 2 – Approximately Maximum‐Likelihood Trees for Large Alignments,” PLoS ONE 5, no. 3 (2010): 9490, 10.1371/journal.pone.0009490. PMC2835736 20224823

[50] I. Letunic and P. Bork , “Interactive Tree of Life (itol) v6: Recent Updates to the Phylogenetic Tree Display and Annotation Tool,” Nucleic Acids Research 52 (2024): W78–W82, 10.1093/nar/gkae268. 38613393 PMC11223838

[51] W. Shen , S. Le , Y. Li , and F. Hu , “SeqKit: a Cross‐Platform and Ultrafast Toolkit for FASTA/Q File Manipulation,” PLoS ONE 11, no. 10 (2016): 0163962, 10.1371/journal.pone.0163962. PMC5051824 27706213

[52] S. Chen , “Ultrafast One‐Pass FASTQ Data Preprocessing, Quality Control, and Deduplication Using fastp,” IMeta 2, no. 2 (2023): 107, 10.1002/imt2.107. PMC10989850 38868435

[53] T. Rognes , T. S. Flouri , B. Nichols , C. Quince , and F. Mahé , “VSEARCH: a Versatile Open Source Tool for Metagenomics,” PeerJ 4 (2016): 2584, 10.7717/peerj.2584. PMC5075697 27781170

[54] R. Leinonen , R. Akhtar , and E. Birney , “The european Nucleotide Archive,” Nucleic Acids Research 39, (2011): D28–D31, 10.1093/nar/gkq967. 20972220 PMC3013801

