# Supplementary material for: MFPD: A Multiple Fungal Pathogen Detection Pipeline Across Diverse Habitats
Source: Adv Sci (Weinh). 2026 Jun 9;13(49):e22660. doi: 10.1002/advs.202522660 (PMC13336447; doi:10.1002/advs.202522660)
Supplement: Supplementary file 1 [file ADVS-13-e22660-s002.docx]

**Supplementary information**

**MFPD: A multiple fungal pathogen detection pipeline across diverse habitats**

Yi Shen^#^, Xinrun Yang^#^, Jiabao Yu^#^, Yaozhong Zhang, Tianjie Yang, Yang Gao, Xiaofang Wang, Alexandre Jousset, Fang-Jie Zhao, Qirong Shen, Gaofei Jiang^*^, Zhong Wei, Yangchun Xu^*^

**Figure S1. Sources of the MFPD database. The MFPD pipeline integrates data from multiple databases: EUKARYOME**^[1]^**, UNITE**^[2]^**, and NCBI.** (a) Venn diagram illustrating species composition across database sources. (b) Pie chart depicting the proportion of sequences contributed by each database source.

**Figure S2. Assessment of sequence missing rate between two ITS subregions.** Each colour denotes to a different fungal phylum.

**Figure S3. Comparison of available ITS subregions under different thresholds in the *in silico* experiment.** (a) Running time for ITS subregion sequences across different variable regions at varying thresholds. (b) Taxonomic assignment accuracy for ITS subregion sequences across different variable regions at varying thresholds. (c) Pathogen detection accuracy using ITS subregions in relative to full-length ITS sequences at varying thresholds. Statistical analyses were performed via the Wilcoxon rank-sum test (*****p* < 0.0001).

**Figure S4. Differences in the relative abundance of pathogens between MFPD and FUNGuild.** (a) Relative abundance of dominant genus-level pathogens in MFPD compared with FUNGuild. (b) and (c) Relative abundance of dominant species-level pathogens in MFPD compared with FUNGuild.

**Figure S5. Comparison of total pathogen abundances and Simpson indices detected by MFPD between healthy and diseased samples from humans (a, d), animals (b, e), and plants (c, f).** (a, d) Healthy and periodontitis samples caused by *Trichophyton rubrum* in humans (Liu et al., 2019)^[3]^. (b) Pathological examples of atopic dermatitis caused by *Malassezia pachydermatis* in animals (Chermprapai et al., 2019)^[4]^. (c) Rhizosphere samples of Fusarium wilt caused by *Fusarium oxysporum* and healthy tomato plants (Xu et al., 2020)^[5]^. Statistical significance was assessed via Wilcoxon rank-sum test.

**Supplementary Reference**

[1]	L. Tedersoo et al., “EUKARYOME: the rRNA gene reference database for identification of all eukaryotes,” Database, vol. 2024, p. baae043, Jun. 2024, doi: 10.1093/database/baae043.

[2]	Abarenkov, Kessy; Zirk, Allan; Piirmann, Timo; Pöhönen, Raivo; Ivanov, Filipp; Nilsson, R. Henrik; Kõljalg, Urmas (2024): UNITE general FASTA release for Fungi 2. Version 04.04.2024. UNITE Community.https://doi.org/10.15156/BIO/2959333

[3]	X. Liu et al., “Characterization of Skin Microbiome in Tinea Pedis,” Indian J. Microbiol., vol. 59, no. 4, pp. 422–427, Dec. 2019, doi: 10.1007/s12088-019-00816-y.

[4]	L. Xu, M. Nicolaisen, J. Larsen, R. Zeng, S. Gao, and F. Dai, “Pathogen Infection and Host-Resistance Interactively Affect Root-Associated Fungal Communities in Watermelon,” Front. Microbiol., vol. 11, p. 605622, Dec. 2020, doi: 10.3389/fmicb.2020.605622.

[5]	S. Chermprapai et al., “The bacterial and fungal microbiome of the skin of healthy dogs and dogs with atopic dermatitis and the impact of topical antimicrobial therapy, an exploratory study,” Vet. Microbiol., vol. 229, pp. 90–99, Feb. 2019, doi: 10.1016/j.vetmic.2018.12.022.
